# Bioavailability of Organic Phosphorus Compounds to the Harmful Dinoflagellate *Karenia mikimotoi*

**DOI:** 10.3390/microorganisms9091961

**Published:** 2021-09-15

**Authors:** Kaixuan Huang, Yanqing Zhuang, Zhou Wang, Linjian Ou, Jingyi Cen, Songhui Lu, Yuzao Qi

**Affiliations:** Research Center of Harmful Algae and Marine Biology, Key Laboratory of Eutrophication and Red Tide Prevention of Guangdong Higher Education Institutes, Jinan University, Guangzhou 510632, China; babyblue02@126.com (K.H.); zzyq7722@163.com (Y.Z.); Wangzhou@126.com (Z.W.); jingyicen@foxmail.com (J.C.); lusonghui1963@126.com (S.L.); tql@jnu.edu.cn (Y.Q.)

**Keywords:** *Karenia mikimotoi*, phosphatase, dinoflagellate

## Abstract

*Karenia mikimotoi* is one of the most well-known harmful bloom species in temperate coastal waters. The present study investigated the characteristics of alkaline phosphatase (APase) and phosphodiesterase (PDEase) activities in hydrolysis of two phosphomonoesters (adenosine triphosphate (ATP) and ribulose 5-phosphate (R5P)) and a phosphodiester (cyclic adenosine monophosphate (cAMP)) in *K. mikimotoi* and compared its growth and physiological responses to the different forms of phosphorus substrates. *K. mikimotoi* produced comparable quantities of APase and PDEase to hydrolyze the organic phosphorus substrates, although hydrolysis of the phosphomonoesters was much faster than that of the phosphodiester. The growth of *K. mikimotoi* on organic phosphorus substrates was comparable to or better than that on inorganic phosphate. The difference in particulate organic nutrients (carbon, nitrogen, and phosphorus) and hemolytic activity supported different rates of hydrolysis-assimilation of the various organic phosphorus substrates by *K. mikimotoi*. The hemolytic activities of *K. mikimotoi* in the presence of organic phosphorus substrates were several times those in the presence of inorganic phosphate during the exponential phase. This suggested the potential important role of organic phosphorus in *K. mikimotoi* blooms.

## 1. Introduction

*Karenia mikimotoi* is one of the most well-known harmful bloom-causing species in temperate coastal waters around the world [1,2,3]. *K. mikimotoi* can produce various toxic substances, such as hemolytic compounds [1,4], cytotoxins [3,5], and reactive oxygen species [3,6], and can thus cause massive mortality of both wild and cultured fish and shellfish. In East Asia, especially in China and Japan, the frequency of *K. mikimotoi* blooms has increased greatly in recent years [7,8]. Blooms caused by *K. mikimotoi* accounted for 65.2% and 31.5% of HAB case numbers associated with fishery damage in China and western Japan, respectively, during 1978–2018 [9]. In China, *K. mikimotoi* blooms have occurred frequently in the coastal waters of Fujian and Zhejiang provinces in the East China Sea since 2002 [8,9]. The most severe damage caused by *K. mikimotoi* ever recorded was reported in the coastal waters of Fujian Province with estimated economic losses exceeding 300 million US dollars in 2012 [3,8]. In 2017, *K. mikimotoi* blooms in the coastal waters of Zhejiang Province lasted for more than one month, affecting an area larger than 1200 square kilometers [10]. The occurrence frequency of *K. mikimotoi* blooms in Japan in 2012–2018 was approximately four times that in 1999–2011 [7]. Blooms of *K. mikimotoi* caused cultured fish mortality with approximate economic losses of 15.3 million US dollars in the Bungo Channel in 2012 [11]. An extensive *K. mikimotoi* bloom caused mass mortality of aquaculture fishes, leading to severe loss in Imari Bay in 2017 [7]. Consequently, numerous investigations have been carried out to study the physiology [12,13,14,15] and toxicology [4,5,6,16,17] of *K. mikimotoi* and to analyze the occurrence mechanism of *K. mikimotoi* blooms [7,11,18,19]. However, *K. mikimotoi* blooms are highly variable in location, timing, duration, and magnitude and do not occur every year, and the ecological processes involved in *K. mikimotoi* bloom formation remain largely obscure.

Nutrient availability is crucial to bloom formation, duration, and magnitude [20]. Some *K. mikimotoi* blooms have been connected to high terrestrial nutrient inputs after rainfall [7,19]. However, there is another contradictory opinion that abundant nutrients might not be necessary for the formation of *K. mikimotoi* blooms [1,3]. In contrast, an environment with relatively low nutrients might benefit *K. mikimotoi* due to competing with other species (such as diatoms) and surviving at relatively low densities, and its strong motility might make this species aggregate in layers allowing outburst under certain conditions [1]. It should be noted that the increasing frequency of *K. mikimotoi* blooms aligns with the varying nutrient structure in the coastal waters of East Asia in recent years. Due to the rapid development of the human population and the concomitant increase in industrial and agricultural activities, the unbalanced input of nitrogen (N) and phosphorus (P) into coastal waters has caused an obvious increase in the N:P ratio in East Asia [21,22]. Phytoplankton normally suffer P deficiency in late spring and summer in this area [21]. Blooms of *K. mikimotoi* have been observed in seawater with a high N:P ratio and a low phosphate concentration [18,23]. Furthermore, *K. mikimotoi* blooms can persist under phosphate-depleted conditions for more than one month [14]. The ability of *K. mikimotoi* to survive and grow under low-phosphate conditions might be crucial to bloom formation and duration.

P is a necessary biogenetic element for phytoplankton growth [24]. The dissolved organic P (DOP) pool is a major contributor to the presence of P in seawater, especially when external phosphate becomes depleted [25]. Phytoplankton that have advantages in utilizing DOP are competitive in phosphate-depleted environments [26]. Phosphomonoesters (PMEs) and phosphodiesters (PDEs) are the most abundant DOP compounds in natural seawater [24]. Previous studies showed that *K. mikimotoi* is capable of utilizing a wide variety of organic P substrates (both PMEs and PDEs) as a sole P source for growth [27,28]. Organic P contributes to outbreaks of *K. mikimotoi* blooms in eutrophic coastal waters [3,27,28]. However, Richardson and Corcoran [12] suggested that axenic *K. mikimotoi* could not utilize PDEs. To date, the characteristics of phosphomonoesterases (PMEases) and phosphodiesterases (PDEases), which play crucial roles in hydrolyzing PMEs and PDEs, respectively [24,26], have been rarely analyzed in terms of *K. mikimotoi*, especially for PDEases. It is uncertain whether *K. mikimotoi* has the relevant phosphatases to hydrolyze both PMEs and PDEs and what the hydrolysis efficiency of various DOP compounds by different phosphatases is in *K. mikimotoi*. In addition, whether different physiological and biochemical responses exist in utilizing various forms of DOP in *K. mikimotoi* is largely unknown. In the present study, *K. mikimotoi* was inoculated onto different P-containing substrates (two PMEs, one PDE, and one inorganic phosphate) as a sole P source. The roles of two extracellular phosphatases (PMEase and PDEase) in the hydrolysis of DOP and the growth and physiological metabolic response (particulate nutrients, pigment contents, and hemolytic activity) of *K. mikimotoi* were studied. The objectives of the study were to analyze the efficiency of phosphatases in hydrolyzing PMEs and PDEs, compare the utilization strategies of *K. mikimotoi* on different forms of P substrates, and demonstrate the potential importance of various organic P sources, thereby explaining the P nutritional physiology and ecology of *K. mikimotoi* blooms.

## 2. Materials and Methods

### 2.1. Culture and Growth Conditions

The *K. mikimotoi* strain (No. HK-5) was maintained in the Algal Collection at the Research Center of Harmful Algae and Marine Biology, Jinan University, China. Prior to the experiment, the cultures were reinoculated 2 times during the exponential phase in an Aquil artificial seawater medium (enriched with f/2) [29], and the phosphate concentration in the medium was gradually reduced from 36 to 6 µmol P L^−1^. The cultures were maintained at 21 ± 1 °C under a light dark cycle of 12:12 h with a photosynthetically active radiation of 100 µmol photon m^−2^ s^−1^. Antibiotics (penicillin G, 200 µg mL^−1^, streptomycin sulfate, 100 µg mL^−1^, and kanamycin sulfate, 100 µg mL^−1^) were added in the medium 48 h before the next inoculation in order to eliminate bacterial contamination. The swimming behavior was checked to ascertain good physiological activity of *K. mikimotoi* using a light microscope (Olympus CX31, Tokyo, Japan). In addition, the cultures were checked for bacterial contamination with 4′,6-diamidino-2-pheny-lindole (DAPI) (Sigma Inc., St. Louis, MO, USA) stain at regular intervals using an epifluorescence microscope (OLYMPUS X61, Tokyo, Japan).

Cells of *K. mikimotoi* in the exponential phase were reinoculated into P-free medium. When the concentration of dissolved inorganic P (DIP) in the medium was below the detection limit (0.10 µmol P L^−1^), the culture was placed in the dark for 4 h and then a LED white light lamp (5W) was placed on the top of the culture. Cells that concentrated on the top layer of the medium by phototaxis were pipetted and diluted with fresh sterile artificial seawater to an initial density of approximately 3.0 × 10^3^ cells mL^−1^. Four P substrates (NaH_2_PO_4_, ATP, ribulose 5-phosphate (R5P), and cyclic adenosine monophosphate (cAMP)) were added separately at a final concentration of 3.0 µmol P L^−1^ in triplicate (hereafter referred to as ‘NaH_2_PO_4′_, ‘ATP’, ‘R5P’, and ‘cAMP’ treatments). Other nutrients were added according to the f/2 medium [30]. Samples for cell density and nutrient analyses were collected every other day, whereas samples to determine the maximum potential quantum efficiency of photosystem II (*Fv/Fm*), two phosphatase activities, particulate nutrients, pigments, and hemolytic activity were collected on days 0, 4, 8, 12, and 16 based on growth curves. Samples were obtained during the hours of 12:00 a.m.–13:00 p.m.

### 2.2. Cell Counts and Fv/Fm Analyses

Cells were fixed in a 2% acid Lugol’s solution. *K. mikimotoi* samples were placed in a Palmer-Maloney counting chamber (0.1 mL) and counted using the light microscope. The specific growth rate (*µ*) of *K. mikimotoi* was calculated according to the equation *µ* = (ln *N*_2_ − ln *N*_1_)/(*t*_2_ − *t*_1_), where *N*_2_ and *N*_1_ are the cell densities at times *t*_2_ and *t*_1_, respectively. For the analysis of *Fv/Fm*, 1 mL of sample was measured with a Phyto-PAM Phytoplankton Analyzer (Walz, Effeltrich, Germany) after dark adaptation for 5 min.

### 2.3. Nutrient Analyses

Seawater was filtered through precombusted (450 °C, 2 h) GF/F filters. The DIP content was determined using the molybdenum blue method described by Valderrama [31]. The total dissolved P (TDP) content was analyzed according to the method of Jeffries et al. [32], employing acid potassium persulfate (K_2_S_2_O_8_). The DOP content was calculated by subtracting the DIP from the TDP. The detection limits of both DIP and DOP were 0.10 µmol L^−1^.

Particulate organic carbon (POC) and particulate organic N (PON) were determined using a CHNS Elementar analyzer (PerkinElmer Series 2200, Boston, MA, USA), whereas particulate organic P (POP) was analyzed according to Solórzano and Sharp [33].

### 2.4. Alkaline Phosphatase (APase) Activity and PDEase Activity Analyses

APase activity (APA) and PDEase activity (PDEA) were measured by monitoring the release of paranitrophenol from 1 mmol L^−1^ paranitrophenylphosphatase (NPP) and 1 mmol L^−1^ bis-NPP, respectively, at 405 nm using a spectrophotometer (Hitachi U-4600, Tokyo, Japan) [34]. Seawater was unfiltered or filtered through 0.22 and 2 µm polycarbonate filters under <100 mm Hg pressure; and, thus, APA and PDEA were divided into three size fractions: free-sized, <0.22 µm, picosized, 0.22–2 µm and nanosized, >2 µm. The subsamples were incubated in the dark at 30 °C for 24 h. Sterile artificial seawater containing different substrates was used as the control. Calibration was performed with the standard solutions of NPP and bis-NPP in the range of 0.01–100 µmol L^−1^.

### 2.5. Pigment Analyses

Aliquots of 30–50 mL of dinoflagellate cultures were filtered through precombusted GF/F filters. Pigments were extracted with *N*,*N*-dimethylformamide in a freezer (−20 °C) for 2 h, and the extracts were filtered through 0.22-μm polycarbonate filters (Millipore, Darmstadt, Germany) to remove cell debris. Pigment concentrations were measured using a Shimadzu LC20A-DAD HPLC system fitted with a 3.5 μm Eclipse XDB C_8_ column (100 × 4.6 mm; Agilent Technologies, Santa Clara, CA, USA) according to the method presented by Zapata et al. [35]. The HPLC system was calibrated with 26 authentic pigment standards from the Danish Hydraulic Institute Water and Environment (Hørsholm, Denmark), which included chlorophyll *a* (Chl *a*), chlorophyll *b*, chlorophyll *c*_2_ (Chl *c*_2_), chlorophyll *c*_3_ (Chl *c*_3_), chlorophyllide *a*, divinyl Chl *a*, magnesium 2,4-divinylpheoporphyrin a_5_ monomethyl ester, pheophytin *a*, pheophorbide *a*, β,β-carotene, β,ε-carotene, alloxanthin, antheraxanthin, 19′-butanoyloxyfucoxanthin, canthaxanthin, diadinoxanthin, diatoxanthin, echinenone, fucoxanthin, 19′-hexanoyloxyfucoxanthin, lutein, neoxanthin, peridinin, prasinoxanthin, violaxathin, and zeaxanthin.

### 2.6. Hemolytic Activity Analyses

The hemolytic activity was analyzed following the method presented by Eschbach et al. [36]. Aliquots of 10 mL of dinoflagellate cultures were centrifuged at 3000× *g* for 10 min. The cells were resuspended in erythrocyte lysis buffer (Eschbach et al., 2001) and ultrasonicated. Fresh red blood cells of Japanese White Rabbits obtained from the Guangzhou Ruite Biological Technology Company (Guangzhou, China) were washed three times with phosphate-buffered saline (pH 7.4) and adjusted to a final concentration of 0.6% (*v*/*v*). Equal volumes of 150 µL of algal samples and erythrocyte suspension were mixed and incubated under an irradiance of 100 µmol photon m^−2^ s^−1^ at 20 °C for 5 h. For the negative control (zero hemolysis), erythrocytes were incubated in phosphate-buffered saline buffer alone. For the positive control (100% hemolysis), erythrocytes were incubated with 1% *v*/*v* Triton X-100. Following incubation, the mixed samples were centrifuged at 3000× *g* for 10 min, and the supernatants were transferred to 96-well plates. Absorption was measured at 414 nm using a microplate reader (HBS-1096B, Detie Co., Ltd., Nanjing, China). The hemolytic activity was expressed as the percentage of hemolysis relative to both the positive and negative controls according to the equation: hemolytic activity (%) = (As − Ab − Aa)/Ac × 100%, where As, Ab, Aa and Ac represent the absorption of the experimental samples (algal samples incubated with erythrocytes), algal samples (algal samples incubated with erythrocyte buffer), negative control, and positive control, respectively.

### 2.7. Data Analysis

One-way ANOVA with Tukey’s test was performed to compare the differences among treatments for each test parameter. A *p* value < 0.05 was considered significant. Prior to the analysis, the data were tested for normality and homogeneity of variation. A Log 10 or square-root transformation of the data was performed prior to any statistical test, when necessary. Statistical analyses were performed using SPSS 19.0 software (SPSS Inc., Chicago, IL, USA).

## 3. Results

### 3.1. DIP and DOP Variations

**Figure 1:**
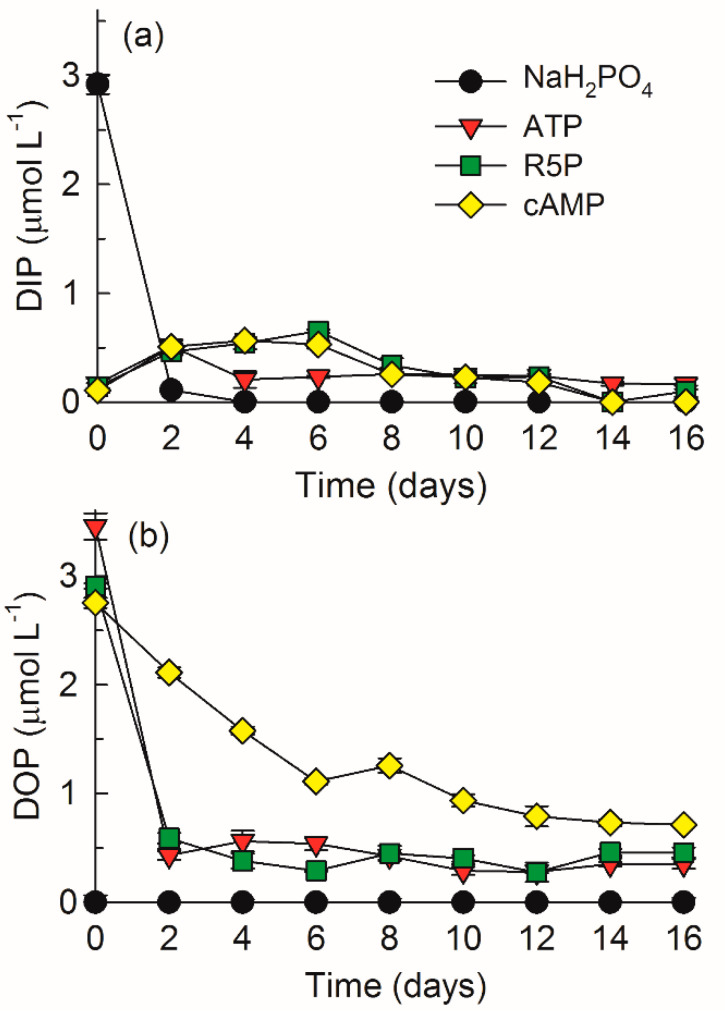
Concentrations of dissolved inorganic phosphorus (DIP) (**a**) and dissolved organic phosphorus (DOP) (**b**) in the NaH_2_PO_4_, adenosine triphosphate (ATP), ribulose 5-phosphate (R5P), and cyclic adenosine monophosphate (cAMP) treatments. The values are the mean ± S.D. (*n* = 3).

DIP decreased sharply from 2.92 ± 0.09 µmol L^−1^ to approximately 0.10 µmol L^−1^ in the first two days and was below the detection limit thereafter in the NaH_2_PO_4_ treatment (Figure 1a). DIP increased to 0.51–0.65 µmol L^−1^ in the first few days and then decreased to lower than 0.10–0.20 µmol L^−1^ after day 12 in the ATP, R5P, and cAMP treatments.

DOP was undetectable in the NaH_2_PO_4_ treatment during the whole study (Figure 1b). DOP decreased sharply from 3.46 ± 0.12 µmol L^−1^ and 2.91 ± 0.02 µmol L^−1^ to approximately 0.50 µmol L^−1^ in the first two days and fluctuated thereafter in the respective ATP and R5P treatments. In contrast, in the cAMP treatment, DOP decreased gradually from 2.75 ± 0.05 µmol L^−1^ at the beginning to 0.71 ± 0.01 µmol L^−1^ at the end of the experiment.

### 3.2. Growth and Fv/Fm

**Figure 2:**
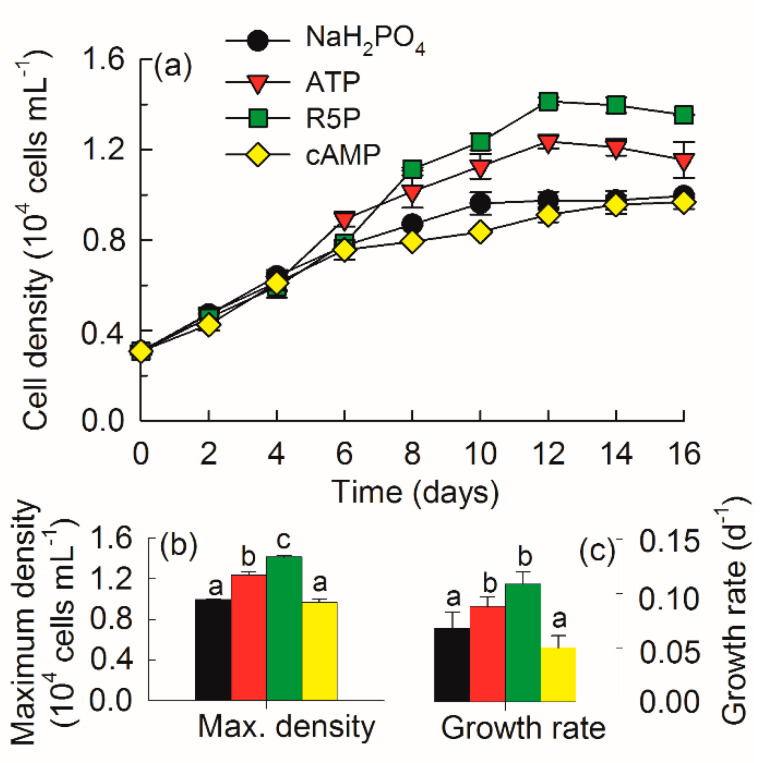
Growth curve (**a**), maximum cell density (**b**), and average growth rate (*µ*) (**c**) of *Karenia mikimotoi* in the NaH_2_PO_4_, adenosine triphosphate (ATP), ribulose 5-phosphate (R5P), and cyclic adenosine monophosphate (cAMP) treatments. The values are the mean ± S.D. (*n* = 3). Significant differences (*p* < 0.05) among different phosphorus substrates are shown with different lowercase letters.

*K. mikimotoi* cells grew slowly and entered the stationary phase on days 10, 12, 12, and 16 in the NaH_2_PO_4_, ATP, R5P, and cAMP treatments, respectively (Figure 2a). The maximum density in the NaH_2_PO_4_ treatment was comparable to that in the cAMP treatment (*p* > 0.05) but was significantly lower than those in the ATP and R5P treatments (*p* < 0.01) (Figure 2b). The average *µ* varied between 0.05–0.12 d^−^^1^, with much higher values in the R5P and ATP treatments (*p* < 0.05) (Figure 2c).

**Figure 3:**
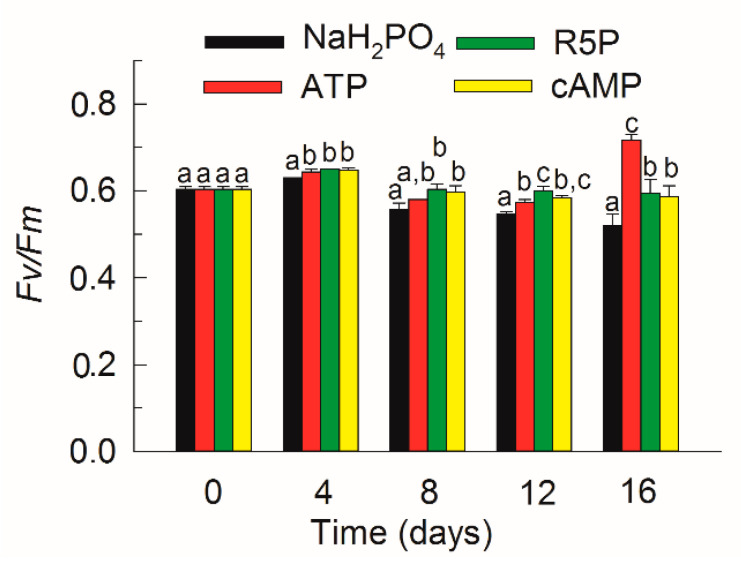
*Fv/Fm* of *Karenia mikimotoi* in the NaH_2_PO_4_, adenosine triphosphate (ATP), ribulose 5-phosphate (R5P), and cyclic adenosine monophosphate (cAMP) treatments on days 0, 4, 8, 12, and 16. The values are the mean ± S.D. (*n* = 3). Significant differences (*p* < 0.05) among different phosphorus substrates are shown with different lowercase letters.

*Fv/Fm* varied between 0.52–0.72 during the whole study among all treatments (Figure 3). *Fv/Fm* in the NaH_2_PO_4_ treatment was significantly lower than that in any other DOP treatment after day 4 (*p* < 0.05). Among the DOP treatments, *Fv/Fm* in the ATP treatment increased markedly and reached the highest value on day 16 (*p* < 0.01).

### 3.3. APA and PDEA

**Figure 4:**
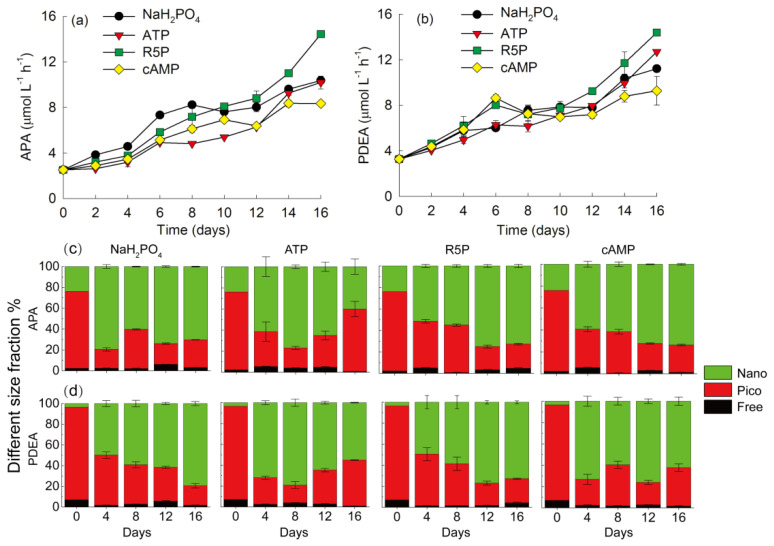
Alkaline phosphatase activity (APA) (**a**) and phosphodiesterase activity (PDEA) (**b**) and different size fractions of APA (**c**) and PDEA (**d**) of *Karenia mikimotoi* in the NaH_2_PO_4_, adenosine triphosphate (ATP), ribulose 5-phosphate (R5P), and cyclic adenosine monophosphate (cAMP) treatments. APA and PDEA are divided into three size fractions: free-sized, <0.22 µm, picosized, 0.22–2 µm, and nanosized, >2 µm. The values are the mean ± S.D. (*n* = 3).

Total APA increased gradually in all treatments but showed some difference in the increasing trends (Figure 4a). The total APA in the NaH_2_PO_4_ treatment was significantly higher than that in any other treatment before day 8 (*p* < 0.05), but the total APA in the R5P treatment was the highest thereafter, especially in the final days (*p* < 0.05). The total APA decreased in the order of NaH_2_PO_4_ ≥ R5P ≥ cAMP > ATP on day 8, whereas the order changed to R5P > NaH_2_PO_4_ = ATP > cAMP on day 16. Free-sized APA accounted for less than 10% of the total APA in all treatments during the whole experiment (Figure 4c). Picosized APA contributed 74% of the total APA on day 0. However, nanosized APA increased greatly and contributed 50–80% of the total APA during the later period, except for a value of 40% in the ATP treatment on day 16.

The values of PDEA were comparable to those of APA during the experiment. The total PDEA in the NaH_2_PO_4_, ATP, and R5P treatments increased gradually with time, whereas the total PDEA in the cAMP treatment reached its first peak on day 6, decreased by 6–20% on days 8–12 and then increased to its peak value on days 14 and 16 (Figure 4b). The total PDEA in the cAMP and R5P treatments was comparable (*p* > 0.05) but was 1.3–1.4 times those in the NaH_2_PO_4_ and ATP treatments (*p* < 0.01) on day 6. At the end of the experiment, the total PDEA in the cAMP treatment was significantly lower than that in any other treatment (*p* < 0.05). Additionally, the total PDEA in the NaH_2_PO_4_ treatment was significantly lower than that in the R5P treatment (*p* < 0.01). Free-sized PDEA accounted for 1–7% of the total PDEA during the whole experiment (Figure 4d). Picosized PDEA contributed 89% of the total PDEA on day 0, but nanosized PDEA was the greatest contributor thereafter (varying between 48–84%).

### 3.4. Particulate Nutrients

**Figure 5:**
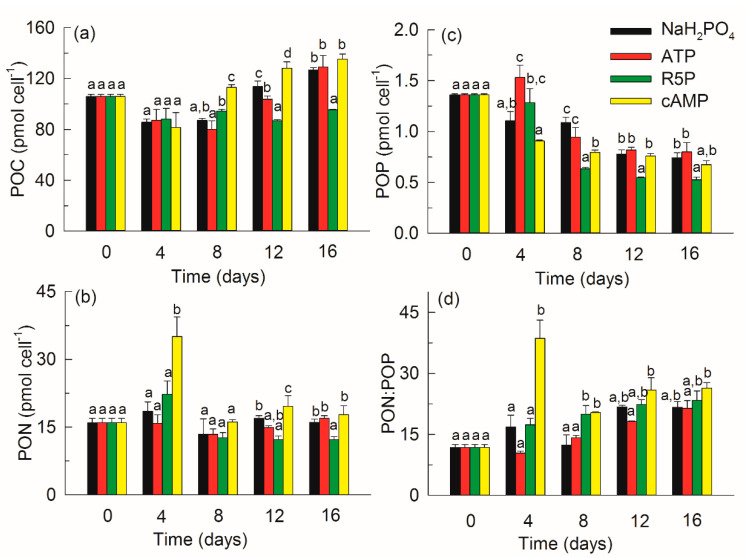
Particulate organic carbon (POC) (**a**), particulate organic nitrogen (PON) (**b**), particulate organic phosphorus (POP) (**c**), and PON:POP ratio (**d**) of *Karenia mikimotoi* in the NaH_2_PO_4_, adenosine triphosphate (ATP), ribulose 5-phosphate (R5P), and cyclic adenosine monophosphate (cAMP) treatments on days 0, 4, 8, 12, and 16. The values are the mean ± S.D. (*n* = 3). Significant differences (*p* < 0.05) among different phosphorus substrates are shown with different lowercase letters.

POC varied from 80.3 ± 6.5 to 135.2 ± 4.1 pmol C cells^−1^ among the treatments (Figure 5a). POC decreased by 20–30% on day 4 and showed no difference among the treatments. Thereafter, POC increased markedly with time except in the R5P treatment. The POC in the cAMP treatment was significantly higher than that in any other treatment on days 8 and 12 (*p* < 0.01). On day 16, the POC in the NaH_2_PO_4_, ATP and cAMP treatments was comparable but was 1.3–1.4 times that in the R5P treatment (*p* < 0.01).

PON varied from 13.4 ± 1.2 to 35.1 ± 4.3 pmol N cells^−1^ among the treatments (Figure 5b). PON increased greatly in the cAMP treatment on day 4, with a value 1.6–2.2 times that in the NaH_2_PO_4_, ATP, and R5P treatments (*p* < 0.01), but decreased markedly to become comparable with the other treatments on day 8. The PON in the R5P treatment was the lowest compared with the other treatments on days 12 and 16.

POP varied from 0.53 ± 0.02 to 1.53 ± 0.12 pmol P cells^−1^ among the treatments (Figure 5c). POP decreased gradually with time except in the ATP treatment on day 4. The POP in the ATP treatment was approximately 1.2–1.7 times that in any other treatment on day 4. In the later period, the POP values in the NaH_2_PO_4_, ATP and cAMP treatments were comparable but significantly higher than that in the R5P treatment (*p* < 0.01).

The initial PON: POP ratio was 11.8 (Figure 5d). The PON:POP ratio in the cAMP treatment increased sharply to 38.6 ± 4.5 on day 4, and then decreased to 20.4 ± 0.2 on day 8. In the later period, the PON:POP ratios in all treatments increased gradually. The PON:POP ratios in the R5P and cAMP treatments were comparable but significantly higher than that in the ATP treatment (*p* < 0.05).

### 3.5. Pigments

**Figure 6:**
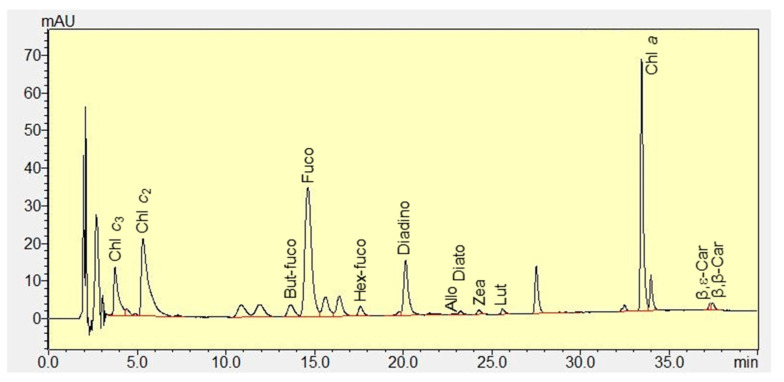
Pigment profiles of *Karenia mikimotoi* analyzed through HPLC. A total of 13 pigments is observed: chlorophyll *c*_3_ (Chl *c*_3_), chlorophyll *c*_2_ (Chl *c*_2_), 19′-butanoyloxyfucoxanthin (But-fuco), fucoxanthin (Fuco), 19′-hexanoyloxyfucoxanthin (Hex-fuco), diadinoxanthin (Diadino), alloxanthin (Allo), diatoxanthin (Diato), zeaxanthin (Zea), lutein (Lut), chlorophyll *a* (Chl *a*), β,ε-carotene (β,ε-Car), and β,β-carotene (β,β-Car).

**Figure 7:**
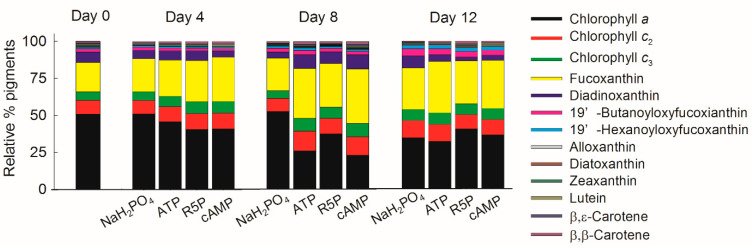
Pigment composition of *Karenia mikimotoi* in the NaH_2_PO_4_, adenosine triphosphate (ATP), ribulose 5-phosphate (R5P), and cyclic adenosine monophosphate (cAMP) treatments on days 0, 4, 8, and 12.

**Figure 8:**
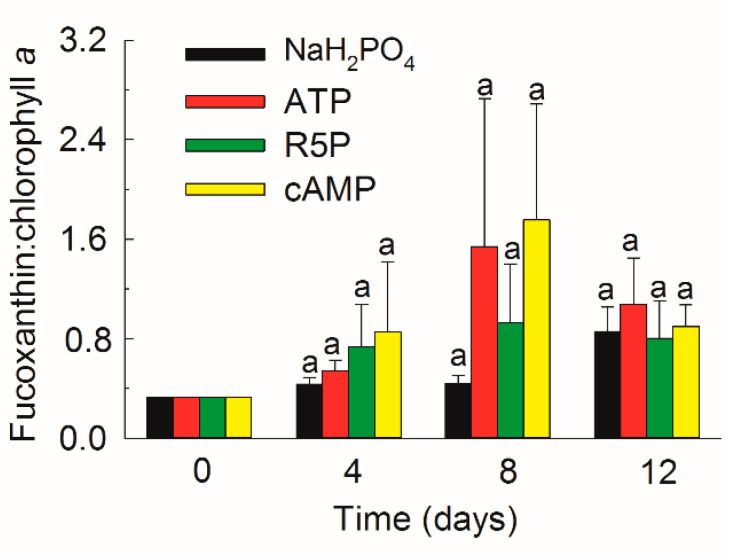
Ratio of fucoxanthin:chlorophyll *a* in the NaH_2_PO_4_, adenosine triphosphate (ATP), ribulose 5-phosphate (R5P), and cyclic adenosine monophosphate (cAMP) treatments on days 0, 4, 8, and 12. The values are the mean ± S.D. (*n* = 3).

A total of 13 pigments were measured in *K. mikimotoi* (Figure 6). Chl *a*, Chl *c*_2_, and Chl *c*_3_ contributed 44.5–66.6% of the total pigments during the whole experiment (Figure 7). Fucoxanthin (19.9–36.8%) was detected as the most abundant carotenoid in *K. mikimotoi*, followed by diadinoxanthin (2.6–9.7%), 19′-butanoyloxyfucoxianthin (1.5–4.6%), 19′-hexanoyloxyfucoxanthin (0.9–2.7%), β,ε-carotene (0.8–2.2%), and β,β-carotene (0.1–1.0%). Lutein, zeaxanthin, alloxanthin, and diatoxanthin were detected in trace amounts of less than 1%. The diagnostic fucoxanthin:Chl *a* ratio ranged between 0.33–1.75, but showed no difference between treatments on the same day. The fucoxanthin:Chl *a* ratio on day 12 was significantly higher than those observed on days 0, 4, and 8 in the NaH_2_PO_4_ treatment (*p* < 0.05) (Figure 8).

**Figure 9:**
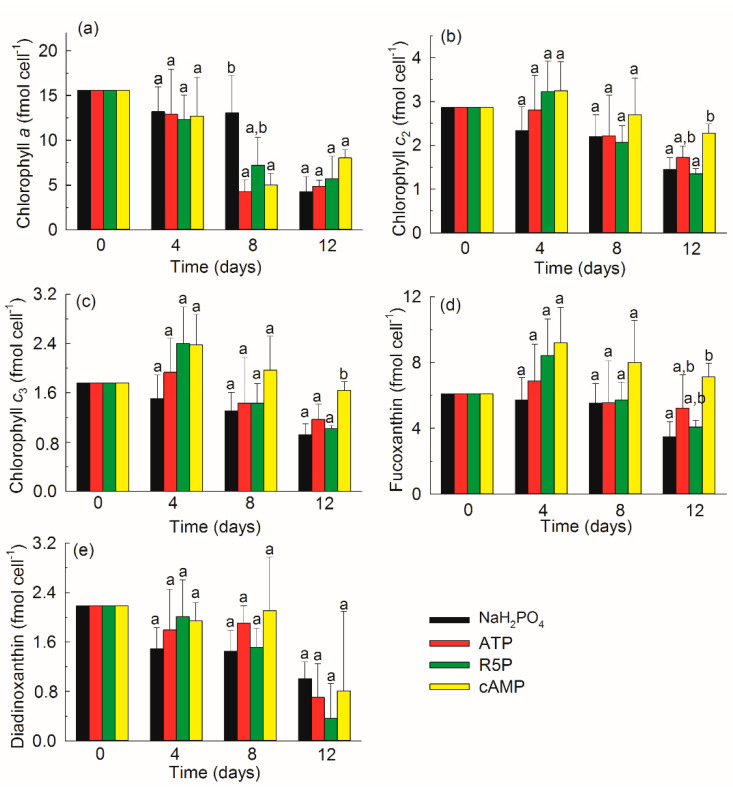
Contents of pigment chlorophyll *a* (**a**), chlorophyll *c*_2_ (**b**), chlorophyll *c*_3_ (**c**), fucoxanthin (**d**), and diatoxanthin (**e**) of *Karenia mikimotoi* in the NaH_2_PO_4_, adenosine triphosphate (ATP), ribulose 5-phosphate (R5P), and cyclic adenosine monophosphate (cAMP) treatments on days 0, 4, 8, and 12. The values are the mean ± S.D. (*n* = 3). Significant differences (*p* < 0.05) among different phosphorus substrates are shown with different lowercase letters.

The cellular content of Chl *a* decreased significantly with time in all treatments (*p* < 0.01 or <0.05) (Figure 9). Additionally, chlorophyll *c*_2_ and chlorophyll *c*_3_ in the NaH_2_PO_4_ and R5P treatments on the initial days 0 and/or 4 were significantly higher than those on day 12 (*p* < 0.01 or < 0.05). The contents of fucoxanthin and diadinoxanthin did not vary with time (*p* > 0.05), except that the fucoxanthin content on day 4 was approximately twice that on day 12 in the cAMP treatment (*p* < 0.05). At most times, the pigment contents did not show differences among the treatments (*p* > 0.05), except that the cellular content of Chl *a* in the NaH_2_PO_4_ treatment was approximately twice that in any other treatment on day 8 (*p* < 0.05) and the cellular contents of Chl *c*_2_, Chl *c*_3_, and fucoxanthin in the cAMP treatment were significantly higher than those in the other treatments on day 12 (*p* < 0.05).

### 3.6. Hemolytic Activity

**Figure 10:**
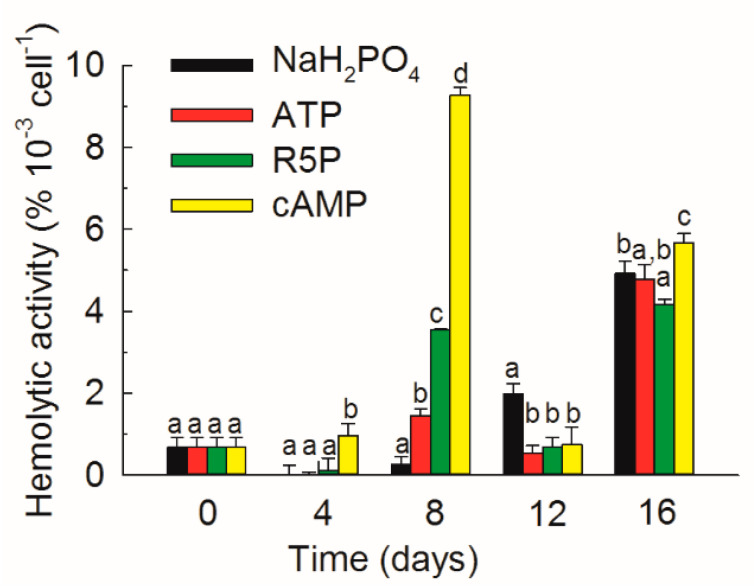
Hemolytic activity of *Karenia mikimotoi* in the NaH_2_PO_4_, adenosine triphosphate (ATP), ribulose 5-phosphate (R5P), and cyclic adenosine monophosphate (cAMP) treatments on days 0, 4, 8, 12 and 16. The values are the mean ± S.D. (*n* = 3). Significant differences (*p* < 0.05) among different phosphorus substrates are shown with different lowercase letters.

The hemolytic activity varied greatly among different treatments and with time (Figure 10). The hemolytic activity in the cAMP treatment was significantly higher than that in any other treatment on day 4 (*p* < 0.01). The difference in hemolytic activity among different treatments increased on day 8, and the activity in the cAMP treatment was 2.6, 6.4, and 36.6 times that in the NaH_2_PO_4_, ATP and R5P treatments, respectively. The hemolytic activity in the DOP substrates decreased, whereas that in the NaH_2_PO_4_ treatment increased on day 12. The activity in the NaH_2_PO_4_ treatment was 2.7–3.7 times that in any other treatment (*p* < 0.001) on day 12. However, the hemolytic activity increased again in all of the treatments, and the activity was the highest in the cAMP treatment and the lowest in the R5P treatment (*p* < 0.05) on day 16.

## 4. Discussion

Blooms of *K. mikimotoi* are often observed in seawater with a high N:P ratio and low phosphate concentration in late spring and summer in the East China Sea [18,23]. The potential of *K. mikimotoi* to utilize DOP as a substitute P source might be crucial to bloom formation and persistence. The DOP pool in seawater is composed of various compounds with different chemical properties, among which P-esters (PMEs and PDEs) and phosphonates appear to account for 75% and 25%, respectively [25,37]. To date, phosphonates have been shown to be available only to heterotrophic bacteria and some cyanobacteria [26,38]. In the P-ester pool, PMEs are normally the most abundant and available DOP [24,37,39]; however, the concentrations of PDEs are comparable or even higher than those of PMEs in some coastal waters [25,40,41]. In the present study, the bioavailability of different forms of PMEs (R5P and ATP) and PDE (cAMP) for the growth of *K. mikimotoi* was compared with that of inorganic NaH_2_PO_4_. The results indicated that *K. mikimotoi* successfully utilized the various organic P compounds for growth, which coincided with previous studies [27,28]. It is worth noting that the growth of *K. mikimotoi* on DOP compounds was comparable to or even better than that on inorganic NaH_2_PO_4_ (Figure 2b,c and Figure 3). An environment with limited phosphate but abundant labile DOP might benefit the growth of *K. mikimotoi*. In addition, the present study also suggested that *K. mikimotoi* is a P storage strategist and is acclimated to external low-phosphate conditions through the efficient utilization of the internal P pool. *K. mikimotoi* is a slow-growing species [3] and had an average *µ* lower than 0.1 d^−1^ in the present study. The sharp decrease in DIP in the NaH_2_PO_4_ treatment in the first two days indicated a luxury uptake of phosphate by *K. mikimotoi* (Figure 1a). *K. mikimotoi* depended on the internal P pool to sustain slow growth and obtained a maximum density on day 10, which was twice that on day 2, and then sustained the density and activity thereafter (Figure 2a and Figure 3). Compared with fast-growing diatoms that burst rapidly in eutrophic environments but also collapse rapidly in oligotrophic environments [42], slow-growing *K. mikimotoi* might be more competitive in environments that vary widely in terms of nutrient availability.

Phosphate is considered the only P compound that can be taken up directly by phytoplankton [24]. Complex DOP compounds should be hydrolyzed by various phosphatases to release phosphate, which is thus available to phytoplankton [24,43]. Differences were observed in the hydrolysis and uptake rates of PMEs and PDE by *K. mikimotoi* in the present study. The hydrolysis rates of ATP and R5P were comparable but much faster than that of cAMP (Figure 1b), suggesting a longer turnover time of the PDE than the PMEs. This result is explainable, since PDEs should first be degraded by the action of PDEase, and, subsequently, the produced PMEs should be degraded by the action of PMEase [37,40]. PDEs are considered biologically more stable than PMEs [25,41]. However, this characteristic of PDEs does not include some biologically labile compounds, such as dissolved DNA that can be cycled in <1 day [40,43]. In the present study, phosphate hydrolyzed from organic P substrates by phosphatases was quickly consumed by *K. mikimotoi*. A small increase in the amount of DIP observed in the organic P treatments during days 2–6 suggested that the hydrolysis rates of DOP compounds were even higher than the uptake rate of phosphate (Figure 1a). Organic P compounds were efficiently hydrolyzed.

In natural seawater environments, there are three important classes of P-esterases: APase, 5′-nucleotidase and PDEase [26,37]. Both APase and 5′-nucleotidase can hydrolyze PMEs, and the difference is that APase is nonspecific for a wide range of PMEs, whereas 5′-nucleotidase acts specifically on 5′-nucleotides [24]. To date, APase is probably the most well-studied phosphatase in both field studies and laboratory species studies [26,38,44]. Compared with APase, PDEase is not as well studied but is also likely important in the cycling of PDEs (DNA, RNA, cyclic nucleotides, and lipids) [39,41]. To the best of our knowledge, the present study is the first to investigate the activities of both APase and PDEase in *K. mikimotoi*. The value of PDEA was comparable to that of APA (Figure 4a,b), suggesting the potential importance of PDEase in hydrolyzing the DOP pool. Bacteria and eukaryotic phytoplankton are the main producers of phosphatases in nature [45]. Richardson and Corcoran [12] found that nonaxenic *K. mikimotoi*, rather than axenic *K. mikimotoi*, could utilize PDEs for growth and suggested that *K. mikimotoi* does not produce PDEase. In the present study, although antibiotics were added in order to eliminate bacteria, the observed picosized phosphatase activities indicated that bacteria still played a role in the hydrolysis of DOP (Figure 4c). However, both nanosized phosphatase activities increased greatly during the whole experiment and contributed more than 65% of the total phosphatases on average, suggesting that the studied strain of *K. mikimotoi* could produce not only APase but also PDEase, even considering that some bacteria might have attached to the algal surface. The production of PDEase by eukaryotic phytoplankton has also been observed in some diatoms and raphidophytes [26,37]. In the present study, the relatively high amounts of APA and PDEA produced by *K. mikimotoi* might indicate a more severe P stress status of phytoplankton than bacteria in phosphate-deficient media. Compared with phytoplankton, the P stress threshold of smaller bacteria is much lower [46]. The enzyme 5′-nucleotidase is widely present in phytoplankton [26]. Luo et al. [13] suggested that 5′-nucleotidase, rather than APase, is responsible for ATP hydrolysis in *K. mikimotoi*. Although the activities of 5′-nucleotidase were not measured in the present study, the relatively low values of APA compared with R5P in the ATP treatment (Figure 4a) suggested that not only APase but also 5′-nucleotidase might play roles in the hydrolysis of ATP.

Phosphatases can be divided into constitutive and inducible classes [26,44]. The present study suggested that most APases and PDEases are inducible enzymes that were abundantly expressed by *K. mikimotoi* under P deficiency. In addition, very low percentages (<10%) of both phosphatases in the dissolved fraction were observed in *K. mikimotoi* during the whole experiment (Figure 4c,d), which was quite different from previously reported results in dinoflagellate species, such as three species of *Prorocentrum* [44] and *Alexandrium catenella* [47], which released abundant phosphatase into seawater over time. Thingstad et al. [46] indicated that the liberation of phosphatases to seawater would drive microbes toward pure phosphate competition by preventing hydrolysis-uptake coupling on the cell surface. Thus, maintaining phosphatases on the cell surface is advantageous for *K. mikimotoi*, allowing this species to directly take up hydrolyzed phosphate without intermediate mixing in the phosphate pool.

The results of particulate C, N, and P reflected the capability of *K. mikimotoi* to hydrolyze and assimilate different forms of P substrates in some way. Cellular stoichiometry is believed to match the environmental nutrient supply at low growth rates [48]. On day 4, when the cell growth among different treatments was comparable, the difference in POP in the organic P treatments (Figure 5c) reflected much faster assimilation of ATP and R5P than cAMP. The hydrolysis-uptake rate of *K. mikimotoi* could not meet the P growth demand in the cAMP treatment, and PON:POP increased to 38.6, which is much higher than the P deficiency threshold of 22 [49]. The significant increase in PON in the cAMP treatment on day 4 (Figure 5b) might reflect the acclimation of *K. mikimotoi* to produce more proteins or enzymes rich in N in response to P deficiency. With the progressive hydrolysis of cAMP to PME and phosphate, the P-deficient status of *K. mikimotoi* in the cAMP treatment was somehow alleviated, the cells showed decreased needs for specific proteins or enzymes, and the PON also decreased on day 8. The ratio of particulate C:N:P reflected variations in the cellular compound composition because major cellular biomolecules differ in their C, N, and P contents [50]. Under P deficiency, phytoplankton show reduced contents of ribosomes and RNA, which are rich in P and slow growth [38], and POP decreased greatly in the present study (Figure 5c). Meanwhile, C-rich compounds, such as carbohydrates and triglycerides, are continually synthesized and accumulated by cells under these conditions [50,51], and POC increased in this study, as shown in Figure 5a. In the later period, the much lower content of particulate nutrients in the R5P treatment was due to the higher growth of *K. mikimotoi* compared with that on other P substrates. *K. mikimotoi* successfully assimilated different forms of P substrates, among which R5P might have been the best substrate. In addition, Luo et al. [13] indicated that *K. mikimotoi* might have the capability to directly transport ATP and its hydrolysis nucleotide products (ADP or AMP) into cells. In the present study, the POC and PON in the ATP treatment did not show higher values than those in the R5P treatment during the exponential phase (days 4 and 8) (Figure 5a,b). Thus, the authors suggest that the major way in which *K. mikimotoi* utilizes ATP as a P source is by taking up phosphate released through hydrolysis by APase or 5′-nucleotidase.

*K. mikimotoi* is a common HAB species in coastal waters [1,3]. Knowledge of its pigment composition aids in rapid taxonomic identification and accurate biomass quantification [52,53]. Pigment compositions show differences between species and even strains [51]. In the present study, in addition to Chl *a*, fucoxanthin, 19′-butanoyloxyfucoxanthin, and 19′-hexanoyloxyfucoxanthin, which are considered the major pigments of *K. mikimotoi* [53,54], contents of Chl *c*_2_ and Chl *c*_3_ were observed in the studied strain (Figure 7). In addition, trace amounts of zeaxanthin were observed (Figure 6), which is not common to every strain of *K. mikimotoi* [54]. The ratio between a marker pigment and Chl *a* is widely used to calculate the contributions of phytoplankton groups to total Chl *a* [55]. The ratio of a marker pigment fucoxanthin to Chl *a* varied greatly from 0.33 ± 0.03 to 1.75 ± 0.93 (Figure 8), which was influenced by both P deficiency and the growth phase. Thus, caution should be taken when using this diagnostic ratio.

Most studies analyze the role of N in pigments because N is the main element in light-harvesting apparatuses [51]. The pigment contents of phytoplankton decrease greatly under N deficiency [53,55]. Additionally, the pigment composition of phytoplankton can change greatly with various N compounds [56]. The effects of variations in the P concentration and P form on the pigment composition and content were analyzed in the present study. The Chl *a* content in the NaH_2_PO_4_ treatment on day 8 (Figure 9a), in which *K. mikimotoi* suffered the most severe P stress (data shown for DIP in Figure 1a and APA in Figure 4a), was significantly higher than that in any other treatment, suggesting that *K. mikimotoi* tried to increase its photosynthetic capability to address P deficiency. Some pigments, especially Chl *a*, decreased greatly from the exponential phase to the stationary phase. One explanation is related to the enhanced P stress in the studied strain. Another reason might be that with increasing biomass, the amount of light received by each cell decreases, so the pigment contents also decrease [53]. Different P compounds did not influence the pigment composition or content of *K. mikimotoi* (Figure 7 and Figure 9). The differences in the contents of some accessory pigments (Chl *c*_2_, Chl *c*_3_, and fucoxanthin) in the cAMP treatment on day 12 were due to the relatively slow growth and lower density compared with those in other treatments.

Blooms of *K. mikimotoi* are connected with fish and shellfish mortality [3,6,7]. Hemolytic activity is a key factor in the toxic potential of *K. mikimotoi* [3,4]. A connection between P deficiency and enhanced cell toxicity has been observed in *K. mikimotoi* [57], and some other dinoflagellates, such as *Alexandrium fundyense* [58], and *Karlodinium veneficum* [59]. However, in the present study, it seemed that the different P forms rather than P concentrations determined the amount of hemolytic activity in *K. mikimotoi* during the exponential phase. The hemolytic activity in the presence of organic P substrates, especially the relatively refractory cAMP, was significantly higher than that in the NaH_2_PO_4_ treatment (Figure 10). It is uncertain whether there is coupling between DOP hydrolysis and hemolytic activity. Li et al. [17] indicated that *K. mikimotoi* was more toxic in the exponential phase than the stationary phase. However, in the present study, hemolytic activity reached a high value during the stationary phase on day 16 (Figure 10). One possible explanation is that severe P stress or limitation induced this species to increase hemolytic activity.

## 5. Conclusions

The frequency of *K. mikimotoi* blooms has increased greatly in recent decades in East Asia [9]. Meanwhile, the nutrient structure in coastal waters has changed from N deficiency to P deficiency, especially during the periods of spring and summer when *K. mikimotoi* blooms occur [18,21,23]. The potential advantage of *K. mikimotoi* in utilizing substitute DOP compounds might be crucial in interspecific competition in phytoplankton communities in phosphate-depleted environments.

PMEs and PDEs are the most abundant organic P available to phytoplankton [25,38]. Our studies confirmed that *K. mikimotoi* could produce both APase and PDEase to hydrolyze DOP compounds. In particular, the quantity and response rate of PDEase were comparable to those of APase, suggesting the potential importance of PDEs as available P sources. Furthermore, the growth and photosynthetic activity of *K. mikimotoi* grown on organic P compounds were comparable to or even higher than those of *K. mikimotoi* grown on inorganic phosphate, which suggested that organic P is a good nutrient source for *K. mikimotoi*. *K. mikimotoi* even produced higher hemolytic activity in the presence of DOP compounds. The present study revealed the strong capability of *K. mikimotoi* to utilize organic P compounds through phosphatases, and thus suggested considering the role of organic P in nutrient dynamics when evaluating the underlying mechanism of *K. mikimotoi* blooms.

## Data Availability

The data that support the findings of this study are available from the corresponding author upon reasonable request.
